# Editorial: Women in thrombosis

**DOI:** 10.3389/fcvm.2023.1159938

**Published:** 2023-02-21

**Authors:** Claire S. Whyte, Marina Panova-Noeva

**Affiliations:** ^1^Aberdeen Cardiovascular & Diabetes Centre, Institute of Medical Sciences, School of Medicine, Medical Sciences and Nutrition, University of Aberdeen, Aberdeen, United Kingdom; ^2^Center for Thrombosis and Hemostasis (CTH), University Medical Center of the Johannes Gutenberg University Mainz, Mainz, Germany

**Keywords:** thrombosis, women, anticoagulants, antiplatelet, fibrinolysis

The aim of the Research Topic “*Women in Thrombosis*” was to promote the work of scientists, that identify as women, in the field of thrombosis. Despite the great advancement in medicine and technology, ischemic heart disease and stroke, the two most prominent features of an arterial thrombosis, remain the top causes of death worldwide. Venous thromboembolism (VTE), remains the third leading cardiovascular cause of global health burden contributing to mortality, morbidity, and health economic consequences (1). The relevance of VTE has become ever more evident with constantly increasing incidence of cancer and the ongoing COVID-19 pandemic.

The selected papers in this Research Topic span from mathematical modeling for prediction of thrombotic events, review of antithrombotic therapies, strategies and monitoring to the role of fibrinolysis in COVID-19 and models used to study this process.

Acute myocardial infarction (MI) occurs after rupture of atherosclerotic plaque triggering coagulation and ultimately occlusion of the coronary artery. The influence of the overall coagulation profile in determining the response is not well understood and on average women experience MIs later in life than men (2). Dunster et al. used validated mathematical modeling to investigate the activation and inhibition of coagulation factors to predict individual haemostatic responses. Measurements of pro- and anti-coagulant proteins in the plasma from patients who had suffered a premature MI was input into the mathematical model and compared to gender-matched healthy control data. They found the level of prothrombin alone was not the best predictor of the coagulation response and patients suffering a premature MI had elevated levels of various coagulation factors and lower tissue factor pathway inhibitor. Importantly, the model predicted sex differences in the haemostatic profile with females having a greater propensity for thrombin generation.

Atrial fibrillation is often complicated by an ischemic stroke from a thrombus development in the left atrial appendage (3). Wang et al. investigated the association between the prognostic nutritional index (PNI), an index of serum albumin and lymphocyte count that reflects the immunonutritional status and left atrial appendage thrombus (LAAT) or spontaneous echo contrast (SEC). Patients with non-valvular atrial fibrillation were retrospectively assessed for the presence or absence of LAAT or SEC by 2 experienced echocardiographers. The study showed that PNI was inversely related to LAAT/dense SEC. The authors noted that low PNI, indicating decreased serum albumin and lymphocyte count levels, implies a poor nutritional state, which usually leads to poor clinical outcomes. Wang et al. conclude that prospective studies with a larger number of patients are required to validate these findings.

Peripheral arterial disease (PAD) results from atherosclerotic plaque burden in the lower extremities and predisposes patients to increased risk of major adverse cardiovascular events (MACE). Dual pathway inhibition was recently proposed to be effective in patients with PAD. Espinola-Klein et al. discuss current knowledge on antithrombotic therapy in patients with PAD and provide guidance recommendations on treatment. The authors recommend antithrombotic strategies to reduce the risk of MACE and major adverse limb events in patients with PAD. The individual bleeding risk should be assessed when considering whether treatments incorporate an anticoagulant, antiplatelet or a combination.

Platelet function tests are used to monitor the efficacy of antiplatelet agents and multiple platforms are available to do this. Nakahara et al. compared current available platelet function tests to monitor antiplatelet therapy. The authors found variable agreement between tests and concluded that the gold standard light transmission aggregometry should continue. In addition, the authors conclude that VerifyNow PRU Test (Werfen, Barcelona, Spain), which measures change in light transmission due to agglutination of fibrinogen-coated beads, offers an alternative for monitoring P2Y12-I-response.

Cancer patients have increased risk of VTE, compared to the general population, that importantly impacts patients' clinical outcome and quality of life. In this Research Topic, Porfidia et al. highlight a thrombotic complication in women that have gynecological or breast cancers. This retrospective study compared the effectiveness of a direct oral anticoagulant (Edoxaban) to low molecular weight heparin in catheter-related upper extremity thrombosis (CRT) in these patients. Residual thrombosis at 3 months did not differ between treatment groups and safety profiles were similar indicating that DOACs could be an appealing alternative to parenteral anticoagulation.

In addition to anticoagulant therapy, elastic compression stocking (ECS) is a frequently prescribed treatment to reduce leg complaints in the acute phase of DVT, to prevent the occurrence of post-thrombotic syndrome and the development of venous ulcers in chronic venous disease (CVD) patients (4). Schreurs et al. performed three-round modified Delphi analysis including 56 health care professionals and 7 patients with DVT and CVD to reach consensus on critical issues of ECS therapy. The study achieved high consensus rates (91%) on 19 out of 21 statements particularly on topics targeting interdisciplinary collaboration, use of initial compression therapy for all patients, accessible involvement of the occupational therapist at the start of ECS therapy to enhance the patient's self-reliance, and a tailored ECS treatment duration for DVT patients. The authors conclude that future research should focus on testing the feasibility of these recommendations and cost consequences in daily clinical practice.

Since the start of pandemic, a large body of evidence demonstrated high risk of VTE particularly in patients with severe COVID-19. In addition to COVID-19-associated hypercoagulable state, increased bleeding tendencies have been reported. According to a recent meta-analysis, the incidence of VTE in hospitalized COVID-19 patients was estimated at 17% and major bleeding at 3.9% (5). Marchetti et al., investigated a wide range of hemostatic, coagulation, fibrinolysis and inflammation-related biomarkers in a prospective study of 101 COVID-19 patients admitted at the intensive care and ward units of two hospitals in the Bergamo area in Italy. The investigators registered a total of 53 events, of which 68% were of thrombotic nature and 32% were bleeding events. The study showed increased plasminogen activator-1 (PAI-1) and tissue plasminogen activator (tPA), together with a high neutrophil to lymphocyte ratio associated with thrombotic events, while decreased Factor XIII was associated with increased bleeding events during hospitalization.

Whyte and Mutch provide a comprehensive overview of methods to study fibrinolysis and the development of models to permit the incorporation of physiological flow rates. Thrombi are formed under shear stresses which influences their structural composition, shape and susceptibility to fibrinolysis. Therefore, these models are essential in advancing our knowledge of the cellular and molecular process dictating thrombus resolution in normal and pathological conditions and assessing novel antithrombotic or thrombolytic therapies.

This Research Topic highlights current opinions on antithrombotic therapies, prognostic markers and modeling, methods for monitoring therapeutic interventions and fibrinolysis as well COVID-19 related fibrinolytic dysregulation.

## Author contributions

CSW and MP-N both wrote the manuscript. All authors contributed to the article and approved the submitted version.
